# First do no harm—beware the risk of *therapeutic plasma exchange* in severe COVID-19

**DOI:** 10.1186/s13054-020-03070-7

**Published:** 2020-06-18

**Authors:** Klaus Stahl, Christian Bode, Sascha David

**Affiliations:** 1 Department of Gastroenterology, Hepatology and Endocrinology, Hannover Medical School, Hannover, Germany; 2 Department of Anaesthesiology and Critical Care, University Medicine Bonn, Bonn, Germany; 3 Department of Nephrology and Hypertension, Hannover Medical School, Carl-Neuberg-Str.1, 30625 Hannover, Germany

To the Editor:

With great interest, we read the article by Keith et al. [1] suggesting adjunctive therapeutic plasma exchange (TPE) as a potential novel treatment approach for severe COVID-19. The basis for their hypothesis builds on the observation that patients with deleterious systemic response to severe infections such as sepsis do not usually die from the underlying pathogen itself but rather from the overwhelming pathological host response to it.

While we are only beginning to understand the pathophysiology behind COVID-19, recent evidence points towards SARS-CoV-2-induced endothelial dysfunction [2], micro- and macrovascular thrombosis, and cytokine-mediated hyperinflammation as key players in determining the clinical outcome. A wide range of extracorporeal treatment methods has been examined in classical septic patients to remove harmful mediators that are thought to be involved in such processes. However, the rationale for TPE goes beyond this simple elimination of circulating injurious molecules, since the exchange of plasma might also replace consumed protective factors that are critical to maintain microcirculatory flow (e.g., ADAMTS-13, protein C) [3] and prevent vascular leak (e.g., angiopoietin-1) [4]. If these theoretical considerations and clinical observations will ultimately lead to an improved survival under controlled conditions, will be investigated in a planned RCT in septic shock patients (*EXCHANGE trial*).

With regard to COVID-19, it has been recognized that humoral immunity is of critical importance in clearing SARS-CoV-2, and treatment with convalescent plasma containing viral-specific neutralizing antibodies has even been suggested as a potential treatment in critically ill COVID-19 patients [5]. In this context, we would be very cautious in recommending TPE using plasma from non-specific donors as the procedure itself might remove critically important neutralizing antibodies against SARS-CoV-2. We gained further insight that we would like to share, when we recently performed rescue TPE in a life-threatening situation of a septic COVID-19 patient. We could not only detect SARS-CoV-2-specific IgG and IgA antibodies in the waste bag plasma but did also reduce the circulating amount of antibodies by one log step.

*Primum non nocere*—*first do no harm*—is a fundamental principle to all physicians originating from the Hippocratic Oath, reminding us that in the case of great uncertainty, restraint might be the most appropriate to not harm the patient despite our best intentions. However, employment of TPE with plasma collected exclusively from reconvalescent donors that carry specific neutralizing antibodies might be both effective and safe.

## Authors’ response

Keith P, Day M, Perkins L, Moyer L, Hewitt K, Wells A

We appreciate Dr. Stahl’s insightful letter with valuable information regarding the effect on viral antibodies with therapeutic plasma exchange (TPE) [6]. This is an area of concern, and the findings are important as we gain knowledge of COVID-19 and potential therapies.

The complex host immune response to infection remains a desired target and appears common to COVID-19 [6]. The theoretical effect and safety of TPE in sepsis has been supported by limited reports [7]. Most of these stem from bacterial infection, though Patel demonstrated clinical efficacy and safety in three pediatric patients during the H1N1 pandemic of 2009 [8]. The findings reported by Dr. Stahl are noteworthy, however, and the potential clinical impact must be considered if considering TPE for sepsis with multiple organ failure due to COVID-19.

As the authors note, the effect of TPE goes beyond cytokine removal, also replacing consumed protective factors that are critical to maintain circulatory flow and prevent vascular leak [6]. This makes TPE unique—and perhaps complementary—to other proposed treatments for sepsis such as remdesivir and convalescent plasma. It is our belief that in severe cases of COVID (and other infections), targeted therapy alone may not be sufficient. Autopsy reports of COVID non-survivors have demonstrated severe endothelial injury and widespread microthrombosis in the lungs, supporting the theoretical role of TPE based on the mechanism of action [9].

Theory alone does not justify treatment, and the gold standard remains prospective, randomized, controlled trials. We eagerly await the results of the EXCHANGE trial and applaud the investigators performing this long-overdue study. Apart from prospective RCTs, evidence for treatment may come from other sources and should be considered in the context of the available data. TPE carries a category 3 recommendation for sepsis with multiple organ failure in the 2019 American Society for Apheresis guidelines [10], stating decision-making should be individualized. The clinical challenge, at present, is identifying those patients likely to benefit, with no specific guidance. It is our responsibility to apply medical knowledge to the best of our ability and judgment, while doing no harm. The art of medicine calls for us to consider the risks and benefits of the available scientific evidence so that we fulfill both of these responsibilities.

As we continue to share our experiences, patient care will continue to improve. In the meantime, we must continue to work using our current evidence and guidelines when providing care.

## Data Availability

Not applicable
